# Ivangustin Alleviates Deoxynivalenol-Induced Apoptosis by Regulating FOXO3a Translocation in Porcine Intestinal Epithelial Cells

**DOI:** 10.3390/toxins17040174

**Published:** 2025-04-02

**Authors:** Tae Hong Kang, Sang Su Shin, Tae Hyun Kim, Sang In Lee

**Affiliations:** 1 Department of Animal Science and Biotechnology, Kyungpook National University, Sangju 37224, Gyeongsangbuk-do, Republic of Korea; camwuil27@knu.ac.kr (T.H.K.); sss@knu.ac.kr (S.S.S.); 2 Research Institute for Innovative Animal Science, Kyungpook National University, Sangju 37224, Gyeongsangbuk-do, Republic of Korea; 3 Department of Animal Science, The Pennsylvania State University, University Park, PA 16802, USA; taekim@psu.edu

**Keywords:** apoptosis, deoxynivalenol, ivangustin, natural product

## Abstract

Deoxynivalenol (DON) is a mycotoxin derived from *Fusarium* species. It is commonly found in crops and has a high detection rate in animal feedstuffs. We previously confirmed that apoptosis could be induced by DON through the FOXO3a (Forkhead box 3a) signaling pathway. In this study, to identify a natural compound to mitigate DON-induced apoptosis via FOXO3a, we performed high-throughput screening. We found that ivangustin (IVAN) alleviated DON-induced cytotoxicity. It also decreased DON-mediated apoptosis and the expression levels of apoptosis-associated genes at the mRNA level. Furthermore, treatment with IVAN inhibited FOXO3a from translocating into the nucleus. The results demonstrated the mitigating effects of the natural compound IVAN on DON-induced apoptosis through the FOXO3a signaling pathway. This study focused on elucidating the mechanism underlying damage caused by DON. According to the results of this study, novel alternatives to mitigate DON cytotoxicity may be developed. This study could provide fundamental data for the formulation of mycotoxin alleviation strategies to improve pig productivity.

## 1. Introduction

Deoxynivalenol (DON) is a widely recognized vomitoxin and a type B trichothecene synthesized by *Fusarium* [1]. DON is frequently detected in grains like wheat, barley, and corn, and it has attracted global attention owing to its detection in animal products, including meat, eggs, and milk [2]. The intake of DON-contaminated crops can have harmful effects on livestock. Pigs are especially susceptible to DON exposure [3]. The gastrointestinal tract is primarily affected by the consumption of DON-contaminated feedstuffs. Furthermore, DON can accumulate and be rapidly absorbed in the gastrointestinal tract, which causes vomiting, reproductive performance disorders, feed refusal, and weight loss [4,5]. Symptoms can differ according to the dose of exposure: high exposure induces diarrhea, vomiting, hemorrhage, and circulatory shock, whereas chronic low exposure leads to immunological alterations, decreased weight gain, and anorexia [6]. At the cellular level, DON is cytotoxic and can cause ribotoxic stress and inhibit protein synthesis, leading to cellular damage [7].

Various strategies have been implemented to mitigate cytotoxicity, such as washing, solvent extraction, heating, irradiation, chemical treatment, and mycotoxin binding. However, these methods are not optimal because they can reduce the nutrient content of grains, feed preference, and the bioavailability of minerals, iron, and vitamins [8]. Natural products include chemical compounds that originate from fungal, plant, animal, marine animal, bacterial, and fungal sources and can exert various biological activities [9]. Owing to the various benefits of natural compounds, they have been considered as alternatives to synthetic chemicals in promoting animal health [10]. In terms of animal health, natural compounds can lead to improved thermotolerance, upregulated antioxidant defense, and improved intestinal villosity performance [11]. In addition, several studies have reported the mitigating effects of natural compounds on the cytotoxicity of mycotoxins [12,13]. However, there is a lack of studies on the application of IVAN to mitigate DON-induced cytotoxicity. Therefore, we examined the molecular process of IVAN underlying the alleviation of DON-induced cytotoxicity.

The FOXO transcription factor has four isoforms in mammals, including FOXO1, FOXO3, FOXO4, and FOXO6 [14]. These isoforms regulate various cellular functions, including survival, proliferation, metabolism, and apoptosis [15]. The FOXO3 transcription factor is especially identified as being involved in the control of apoptosis through the transcription regulation of pro- and anti-apoptotic genes [16]. Apoptosis generally serves as a defense mechanism to remove unnecessary or damaged cells; however, excessive apoptosis can result in pathological conditions such as malignancy, infectious diseases, and autoimmune disorders [17,18]. In the small intestinal epithelium, excessive apoptosis can cause the disruption of barrier integrity, which leads to inflammatory diseases through external stimulation [19].

The intestinal epithelium is organized as single-cell layers composed of epithelial cells, which are connected by junctions such as tight junctions, adherence junctions, gap junctions, and desmosomes [20]. In the intestinal epithelium, nutrients, fluids, and external antigens can enter through different pathways owing to the selective permeability of the cells. Essential nutrients and fluids are transported via passive diffusion across the cell membrane or are taken up by the carrier–receptor transcellular pathway; however, external antigens move between the cells, i.e., the paracellular pathway [21]. The intestinal barrier can be disrupted because of pathogen infection, insecticides, food additives, diseases, drug, and mycotoxin exposure. Mycotoxin exposure is a major issue and is well-known to damage the integrity of the intestinal barrier [22].

## 2. Results

### 2.1. Identification of Effective Natural Product Candidates for the Mitigation of DON-Induced Cytotoxicity

**Figure 1:**
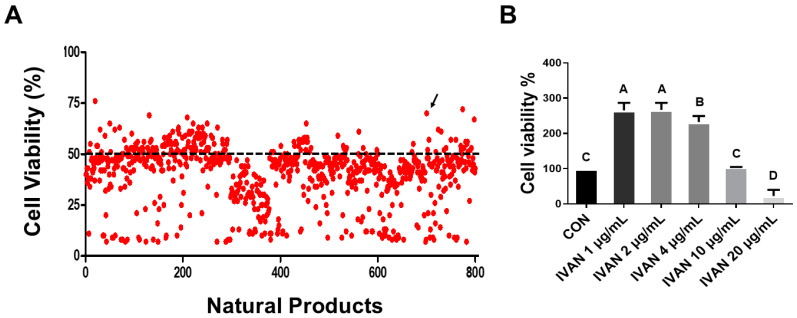
Effect of natural compounds on deoxynivalenol (DON)-induced cytotoxicity. WST-1 was applied to assess cell viability. (**A**) Cells were incubated with 2 μg/mL DON and 2 μg/mL natural compounds for 24 h. The dotted line indicates DON-caused cytotoxicity in cells. The arrow represents the cytotoxicity mitigation effect of IVAN in DON-treated cells. (**B**) Cells were exposed to various concentrations of IVAN (1, 2, 4, 10, and 20 μg/mL) for 24 h. Differences in upper-case letters mean mark differences (*p* < 0.05) among the treatments according to Duncan’s multiple range test. Error bars represent the standard error of analyses conducted in triplicate.

To investigate the effectiveness of natural products on DON-treated cells, WST-1 cell proliferation assay was performed. Our previous study results indicated that the half-maximal inhibitory concentration (IC50) of DON was 2.28 μg/mL for IEPC cells. Therefore, various natural compounds were applied to DON-treated IPEC-J2 cells at 2 μg/mL for a duration of 24 h. Of the 50 natural products, candidates exhibiting mitigating effects on DON-induced cytotoxicity were selected. To confirm the cytotoxicity of IVAN, cells were exposed to different concentrations (1, 2, 4, 10, and 20 μg/mL) for 24 h. The cell proliferation ratio was the highest in the IVAN-treated group compared with the top 1, 2 group when cells were exposed to only natural products. Based on these results, IVAN was selected for subsequent investigation (Figure 1A). The proliferation rate of cells was significantly elevated when exposed to 4 μg/mL IVAN in comparison to the control. In comparison with other treatment groups, the group IVAN at a concentration of 2 μg/mL exhibited the highest increase. In the group treated with 20 μg/mL IVAN, the proliferation rate was significantly decreased (Figure 1B). Based on these results, 2 μg/mL IVAN was used for later studies.

### 2.2. Suppression of DON-Induced Apoptosis by IVAN

**Figure 2:**
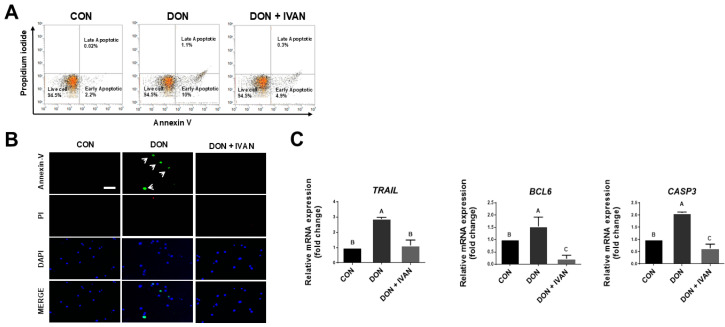
Alleviation of DON-induced apoptosis by IVAN in intestinal porcine epithelial cells. (**A**) FACS analysis and Annexin V/PI staining were conducted to determine the percentage of apoptotic cells. (**B**) Apoptotic cell detection was carried out using Annexin V (green) and PI (red). The arrows indicate Annexin V-positive cells. 4′,6-diamidino-2-phenylindole (DAPI; blue) was used to stain the nuclei. Scale bar = 20 μm. (**C**) The expression levels of apoptosis-related genes (TRAIL, BCL6, and CASP3) at the mRNA level were measured. Differences in upper-case letters mean marked differences (*p* < 0.05) among the treatment groups as determined by Duncan’s multiple range test. Error bars indicate the standard error of analyses conducted in triplicate.

To examine the effect of IVAN on DON-mediated apoptosis, we performed Annexin V/propidium iodide (PI) staining and quantitative reverse transcription polymerase chain reaction (qRT-PCR). Apoptotic rates were measured by flow cytometry analysis and fluorescence microscopy. Following DON exposure, the detection rates of early and late apoptotic cells among IPEC-J2 cells were 10% and 1.1%, respectively. However, when IPEC-J2 cells were treated with both DON and IVAN, these rates were 4.9% and 0.3%, respectively. In addition, apoptotic cells were visualized. The number of early apoptotic IPEC-J2 cells was significantly increased in the DON-treated group compared with the untreated control group. However, the number of apoptotic cells was markedly decreased in the DON- and IVAN-treated groups compared with the DON-exposed group (Figure 2A). Similarly, in comparison to the control group and the DON- and IVAN-treated groups, the DON-treated group showed an increased number of Annexin V-positive cells (Figure 2B). To determine the mRNA levels of apoptosis-associated genes, we conducted qRT-PCR. The expression levels of apoptosis-associated genes (*TRAIL*, *BCL6*, and *CASP3*) were markedly elevated in DON-treated cells in comparison to untreated IPEC-J2 cells. However, their expression levels were markedly decreased when cells were exposed to both DON and IVAN (Figure 2C). These results indicated that IVAN could inhibit apoptosis induced by DON.

### 2.3. Effect of IVAN on DON-Regulated FOXO3a Expression

**Figure 3:**
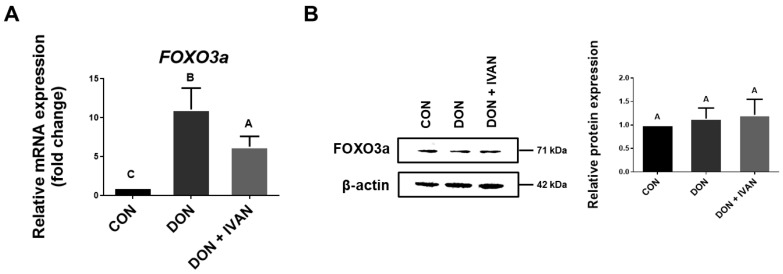
Effect of IVAN on the mRNA and protein expression levels of FOXO3a induced by DON. Cells were exposed to 2 μg/mL DON and IVAN for 24 h. (**A**) The mRNA expression level of *FOXO3a* was compared between untreated IPEC-J2 cells and IPEC-J2 cells treated with 2 μg/mL DON or 2 μg/mL DON and IVAN (n = 3). The error bars denote the standard error based on triplicate analyses. (**B**) FOXO3a protein expression levels were compared between untreated cells and those treated with 2 μg/mL DON alone or with both 2 μg/mL DON and IVAN. Variations in upper-case letters denote notable differences (*p* < 0.05) among the treatment groups as determined by Duncan’s multiple range test. Error bars represent the standard error of analyses conducted in triplicate.

To determine the expression levels of FOXO3a at both the mRNA and protein levels, qRT-PCR and western blot assay were performed. The level of FOXO3a mRNA expression was notably increased in the DON-exposed group relative to the control. However, the level of FOXO3a mRNA expression was noticeably decreased in the group treated with DON and IVAN as opposed to the DON-exposed group (Figure 3A). Conversely, the protein level of FOXO3a exhibited no significant difference between the groups (Figure 3B).

### 2.4. Inhibition of the DON-Mediated Translocation of FOXO3a into the Nucleus by IVAN

**Figure 4:**
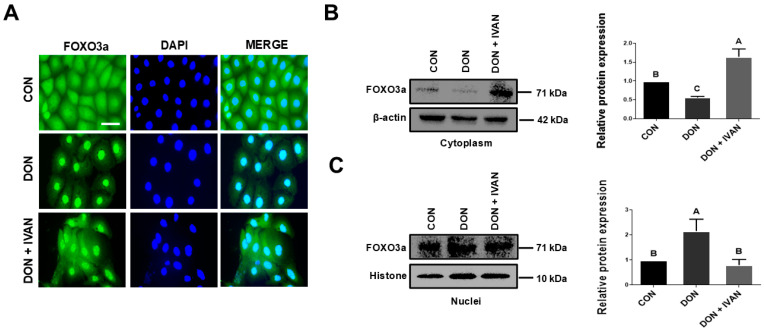
Inhibition of DON-mediated FOXO3a translocation from the cytoplasm into the nucleus by IVAN. (**A**) Translocation of FOXO3a was measured by immunocytochemistry. DAPI was utilized to stain the nuclei. Scale bar = 40 μm. (**B**) Protein expression levels in the cytoplasm were measured. (**C**) Protein expression levels in the nuclei were measured. Differences in upper-case letters mean mark differences (*p* < 0.05) among the treatments according to Duncan’s multiple range test. Error bars represent the standard error of analyses conducted in triplicate.

To examine the impact of IVAN on the translocation of FOXO3a triggered by DON, immunocytochemistry and western blot assay were performed. In untreated IPEC-J2 cells, the expression of FOXO3a was identified in the cytoplasm and nucleus. Upon treatment with DON, FOXO3a expression was only identified in the nucleus (Figure 4A). Western blot assay was performed after obtaining protein extracts from the cytoplasm and nucleus. The cytoplasmic FOXO3a protein expression was reduced in the DON-treated group in contrast to the untreated group, whereas its nuclear expression level was elevated. FOXO3a expression levels were higher in the cytoplasm but lower in the nucleus of IPEC-J2 cells exposed to both DON and IVAN (Figure 4B,C). These results revealed that IVAN could suppress the DON-mediated translocation of FOXO3a into the nucleus.

### 2.5. IVAN Inhibits DON-Mediated ERK1/2 Phosphorylation

**Figure 5:**
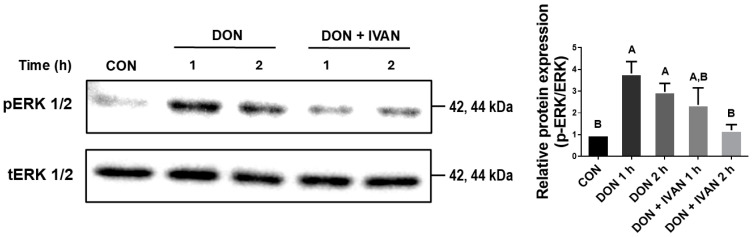
Inhibition of the translocation of FOXO3a by IVAN via ERK1/2 suppression. ERK1/2 phosphorylation levels were measured after treatment with deoxynivalenol (DON) alone or with DON and IVAN for 1 or 2 h at a concentration of 2 μg/mL. Differences in upper-case letters mean mark differences (*p* < 0.05) among the treatments according to Duncan’s multiple range test. Error bars represent the standard error of analyses conducted in triplicate.

To evaluate the effect of IVAN on DON-induced phosphorylation of ERK1/2, western blot assay was performed. ERK1/2 phosphorylation was increased after 1 and 2 h in IPEC-J2 cells exposed to DON relative to the control. On the other hand, when IPEC-J2 cells were exposed to both DON and IVAN for 2 h, ERK1/2 phosphorylation was significantly suppressed (Figure 5). These results indicate that IVAN could inhibit DON-mediated ERK phosphorylation in cells.

## 3. Discussion

DON is the most common mycotoxin found in grains and feed ingredients [23]. It is a secondary metabolite produced by *Fusarium* [24]. The main features contributing to the toxicity of the DON molecule include the C9/C10 double bond, the epoxy group of C12/C13, and the free hydroxyl group [25]. The effect of DON differs depending on whether the exposure is acute or chronic. Acute DON exposure at high doses causes diarrhea, vomiting, and hemorrhage/necrosis of the intestinal tract, whereas chronic DON exposure induces anorexia, reduced weight gain, the reduction of nutritional efficiency, immunotoxicity, and the necrosis of the gastrointestinal tract [26]. At the molecular level, DON exhibits ribotoxicity. It can bind to the 3′-end of the 28S rRNA and induce the ribotoxic stress syndrome and the inhibition of translation, which leads to DON-induced apoptosis, inflammatory responses, oxidative stress, and the disruption of intestinal barrier function by reducing tight junction protein expression [27,28]. Further studies examining the mechanism underlying DON-induced damage are required for a better understanding. We have demonstrated that DON could induce apoptosis [29], and, based on existing data, we performed high-throughput screening to determine a natural product to reduce DON-induced cytotoxicity (Figure 1A,B).

Plant natural compounds and secondary metabolites are produced for protection against insects, microbes, and herbivores. These compounds have a low molecular weight and serve as natural antifungals, antivirals, herbicides, antibacterials, and insecticides [30]. In the livestock industry, feed additive supplementation is a strategic management process used to maintain livestock growth and health. Recently, several studies have emphasized the use of natural compounds from plants as replacements for existing additives [31]. Natural compounds can also be used for the mitigation of mycotoxin toxicity. Phenols, oxygenated terpenoids, and terpenes are known to have antifungal properties. Their functions differ according to the specific structure of the chemical compounds, such as the position and type of substituents [32]. Sesquiterpene lactones (STLs) are secondary metabolites in plants that are commonly found in nature. The α-methylene-γ-lactone moiety of STLs has attracted attention because it exerts biological effects on cancer development and progression. IVAN is a prevalent 6/6/5-tricyclic eudesmane STL and has diverse biological activity, including antifungal, antibacterial, anti-inflammatory, antidiabetic, and insecticidal effects [33,34]. However, research on the impact of IVAN on DON-mediated apoptosis is limited. In a previous study, DON was found to induce apoptosis through the FOXO signaling pathway. Therefore, we performed this study to assess the apoptosis-modulating effects of IVAN on DON-induced apoptosis in IPEC-J2 cells.

**Figure 6:**
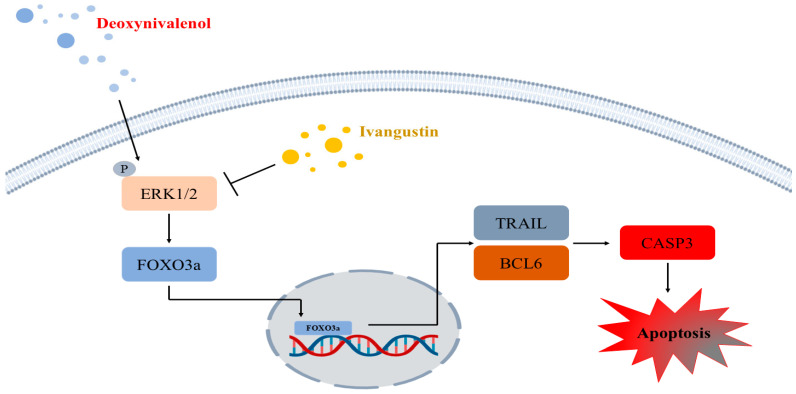
Schematic illustration of the molecular processes by which IVAN suppresses DON-mediated apoptosis in IPEC-J2 cells. DON triggers the phosphorylation of ERK1/2, and FOXO3a is translocated into the nucleus, leading to the transcription of BCL6 and TRAIL. IVAN inhibits FOXO3a translocation into the nucleus and suppresses apoptosis induced by DON.

To confirm the mitigating effects of IVAN on DON-induced apoptosis in IPEC-J2 cells, we performed Annexin V/PI staining and qRT-PCR (Figure 2A–C). In mammals, the FOXO family is composed of four members: FOXO1, FOXO3, FOXO4, and FOXO6, each exhibiting distinct tissue-specific expressions [35]. Specifically, FOXO3a regulates apoptosis, proliferation, DNA damage, tumorigenesis, and cell cycle progression [36]. FOXO3a is a well-known inducer of apoptosis, which is regulated through mitochondria-dependent or -independent pathways [37,38]. The translocation of FOXO into the nucleus triggers apoptosis via the transcription of apoptosis-associated genes such as *TRAIL*, *FasL*, *BCL6*, and the *Bcl-2*-interacting mediator of cell death (*BIM*) [39]. In apoptosis involving the mitochondrial-independent pathway, upregulated *TRAIL* leads to the binding of the death receptor and activates caspase 8, which results in apoptosis. *BCL6* regulates the suppression of *BCL2* family members such as *BIM* and *bNIP3* and results in the induction of mitochondrial permeability, which leads to apoptosis [40]. In our previous study, DON triggered apoptosis and elevated the mRNA levels of genes associated with apoptosis (BCL6, CASP3, and TRAIL). In addition, DON induced FOXO3a to translocate into the nucleus through the ERK1/2 signaling pathway. However, mitigating strategies for DON-mediated damage in IPEC-J2 cells require further studies. To discover new natural products that can regulate the translocation of FOXO3a into the nucleus induced by DON, we performed high-throughput screening. It was found that IVAN decreased DON-induced apoptotic cells, and the expression of genes associated with apoptosis, including TRAIL, BCL6, and CASP3 (Figure 2C). In addition, the translocation of FOXO3a into the nucleus was inhibited by IVAN, and it suppressed the phosphorylation of ERK1/2 upstream from the FOXO signaling pathway (Figure 4, Figure 5 and Figure 6).

## 4. Conclusions

This study examined the mitigating effects of IVAN on DON-mediated apoptosis in IPEC-J2 cells. IVAN inhibited the FOXO3a translocation into the nucleus mediated by DON and reduced the expression levels of the apoptosis-related genes *TRAIL*, *BCL6*, and *CASP3*. These findings could enhance our understanding of the molecular processes through which IVAN alleviates DON-induced apoptosis. Furthermore, this study may serve as foundational research to address the reduction in productivity caused by DON.

## 5. Materials and Methods

### 5.1. Cell Culture and Treatment of Cells

From DSMZ (German Collection of Microorganisms and Cell Cultures, Braunschweig, Germany), the IPEC-J2 cell line was sourced. The culture medium contained Dulbecco’s modified Eagle’s medium (DMEM) (Thermo Fisher Scientific, Wilmington, DE, USA) with 10% fetal bovine serum (FBS) (Thermo Fisher Scientific, Wilmington, DE, USA) and 1% penicillin–streptomycin (Thermo Fisher Scientific, Wilmington, DE, USA). Cells were cultured in a CO_2_ incubator at 37 °C. According to previous cell viability data, DON was administered at a concentration of 2 µg/mL (6.75 µmol/L) [29]. Treatment with IVAN was performed at a concentration of 2 µg/mL (8.05 µmol/L) in cells. All natural compounds were obtained from the Natural Products Bank of the National Institute for Korean Medicine Development (Table 1).

### 5.2. Cell Viability

In 96-well plates, cells were seeded at a density of 1 × 10^4^ cells/100 μL and cultured for 32 h in a CO_2_ incubator at 37 °C. The medium was removed, and the cells were incubated overnight in DMEM. IPEC-J2 cells were exposed to 2 μg/mL DON and natural compounds for 24 h. After treatment with WST-1 reagent for 2 h (Roche Diagnostics GmbH, Mannheim, Germany), cell viability was evaluated using a microplate reader at an absorbance of 450 nm.

### 5.3. Annexin V and PI Staining

After collecting the IPEC-J2 cells, they were rinsed with PBS (Thermo Fisher Scientific, Wilmington, DE, USA) and centrifuged for 5 min at 264× *g*. The supernatant was then discarded. After resuspending the cell pellet in annexin binding buffer, 5 μL of Annexin V (Thermo Fisher Scientific, Wilmington, DE, USA) and 100 μg/mL of PI were added and incubated for 15 min in the dark. The BD FACSVerse flow cytometry (BD Biosciences, San Jose, CA, USA) was utilized for analysis. DAPI staining was performed on the cells (Vector Laboratories, Burlingame, CA, USA) and stained cells were observed using fluorescein microscopy (Korealabtech, Seongnam-si, Republic of Korea).

### 5.4. Extraction of Nuclear and Cytoplasmic Proteins

The NE-PER Nuclear (NER) and Cytoplasmic Extraction Reagents (CER) were used to extract cytoplasmic and nuclear proteins (Thermo Fisher Scientific, Wilmington, DE, USA). In brief, after the cells were harvested, they were rinsed with cold PBS and centrifuged at 500× *g* for 3 min. The PBS was removed, and CERI was applied to the cell pellet, followed by vortexing for 15 s and incubation for 10 min on ice. Additional CERII reagent was added to the suspension, incubated for 1 min, and centrifuged for 5 min at 16,000× *g*. The supernatant was moved to a pre-chilled microtube as the extracted cytoplasmic protein portion, and NER was applied to the remaining pellet, followed by vortexing for 15 s every 10 min for 40 min. The suspension was centrifuged for 10 min at 16,000 × *g*. The supernatant was transferred to a cold microtube as the extracted nuclear protein portion.

### 5.5. Real-Time Quantitative PCR and Western Blot Assay

Total RNA was isolated with the AccuPreP Universal RNA Extraction kit (BioNEER, Daejeon, Republic of Korea). Then, 1 μg of RNA was utilized to synthesize cDNA with the DiaStar™ RT Kit (SolGent, Daejeon, Republic of Korea). For the amplification of genes using PCR, Primer3 (primer3.ut.ee) was used for primer design. The following protocol was used for qRT-PCR: 95 °C for 3 min, followed by 40 cycles at 95 °C for 15 s, 55–58 °C for 15 s, and 72 °C for 15 s. The target gene levels were calculated using the 2^−ΔΔCt^ method, with results normalized to GAPDH expression. The primer sequences that were used are shown in Table 2.

For the protein extraction process, lysis buffer was applied to the cells, and a protease inhibitor was included in the buffer. The concentration was determined through the BCA Protein Assay (Thermo Fisher Scientific, Wilmington, DE, USA). Subsequent to electrophoresis on a 9% polyacrylamide gel for 1 h at 100 V, the proteins were transferred to a membrane and blocked for 1 h. The membrane underwent overnight incubation with anti-ERK1/2 (Cell Signaling Technology, Danvers, MA, USA) and anti-FOXO3a (Novus Biologicals, Centennial, CO, USA) primary antibodies. Following three washes with PBS, the membrane was exposed to the secondary antibody for 1 h. The ChemiDoc imaging system was utilized to visualize the protein bands.

### 5.6. Immunofluorescence Staining

Cells were exposed to 4% paraformaldehyde for 15 min at room temperature and subsequently washed three times with PBS, each wash lasting 5 min. Following a 1 h blocking step (1× PBS, 5% normal goat serum, 0.3% triton X-100), the cells were incubated overnight at 4 °C with a primary antibody solution (1× PBS, 0.3% triton X-100, 1% BSA). Following a 1 h incubation with a second antibody, the cells were stained with DAPI (Vector Laboratories, Burlingame, CA, USA). A fluorescence microscope was used to obtain images (Korealabtech, Seongnam-si, Republic of Korea).

### 5.7. Statistical Analysis

Statistical analysis was performed using the general linear model (PROC-GLM) in SAS 9.4 to compare the control and treatment groups. The error bars represent the standard error based on analyses conducted in triplicate. Results are shown as the mean ± standard error of the mean (n ≥ 3, where n indicates the number of replicates). A *p*-value of less than 0.05 was considered statistically significant. Duncan’s multiple range test was applied to determine differences.

## Figures and Tables

**Table 1:** List of top-50 natural compounds.

Material Name	Chemical Formula	Molecular Weight	Cell Viability (%)
Procyanidin B3	C_30_H_26_O_12_	578.52	76
Bavachromanol	C_20_H_20_O_5_	340.37	74
Ivangustin	C_15_H_20_O_3_	248.32	72
Santamarine	C_15_H_20_O_3_	248.14	71
(+)-Catechin	C_15_H_14_O_6_	292	70
Coniferaldehyde	C_10_H_10_O_3_	178.06	70
Lucidine primeveroside	C_26_H_28_O_14_	564.49	69
2-Stearo-1,3-dilinolein	C_53_H_102_O_6_	874.76	68
Xanthalongin	C_15_H_20_O_3_	248.32	67
Fargesin	C_21_H_22_O_6_	370.4	65
8,8′-Bieckol	C_36_H_22_O_18_	742	65
Dauricumine	C_19_H_24_CINO_6_	397.87	65
trans-(R)-Resveratrol	C_14_H_12_O_3_	228.08	65
(+)-Catechin-7-O-β-D-apio furanoside	C_20_H_22_O_10_	422	64
6,9-Epi-8-O-acetylshanziside methyl ester	C_19_H_28_O_12_	448	63
(2S)-4′,6-Dihydroxy-7-methoxyflavan	C_16_H_16_O_4_	272.29	63
Astragaloside I	C_45_H_72_O_16_	868.48	63
Chiratenol	C_30_H_5_0O	426.73	63
Hispidin	C_13_H_10_O_5_	246.22	63
24-Methylenecycloartanol	C_31_H_52_O	440.4	63
Glucoaurantio-obtusin	C_23_H_24_O_12_	492.43	62
Euphorbia Factor L2	C_38_H_42_O_9_	642	62
Spirobenzofuran	C_15_H_18_O_4_	262	62
Bavacoumestan C	C_20_H_16_O_7_	368.34	62
Kaempferol	C_15_H_10_O_6_	286.2	62
Benzoylpaeoniflorin	C_30_H_32_O_12_	584.57	61
4′-Hydroxydehydrokawain	C_14_H_12_O_4_	244	61
6,6′-Bieckol	C_36_H_22_O_18_	742	61
2-Benzyl-2,3′,4′,6-tetrahydroxybenzo[b]furan-3(2H)-one	C_15_H_12_O_6_	288.26	61
Germacrone epoxide	C_15_H_22_O_2_	234	60
(2S)-4′, 6-Dihydroxy-7-methoxyflavanone	C_16_H_14_O_5_	286.27	60
3-epi-Oleanolic acid	C_30_H_48_O_3_	456.7	60
5,5′-Dihydroxy-7,8-dimethoxyflavanone-2-O-b-D-glucopyranoside	C_23_H_26_O_12_	494.14	60
Apiopaeonoside	C_20_H_28_O_12_	460	60
Astragaloside III	C_41_H_68_O_14_	784.46	60
Cimifugin	C_16_H_18_O_6_	306.31	60
8-O-acetyl-harpagide	C_17_H_26_O_11_	406.39	59
Sophoraflavanone G (vexibinol)	C_25_H_28_O_6_	424.18	59
Germacrone	C_15_H_22_O	218	59
Brazilein	C_16_H_12_O_5_	284.26	59
2-(3-Hydroxy-2-oxoindolin-3-yl) acetic acid	C_10_H_9_NO_4_	207	59
Kalopanaxsaponin C	C_65_H_106_O_31_	1382.67	58
Hederacholichiside F	C_65_H_106_O_31_	1383.52	58
Ferulic acid	C_10_H_10_O_4_	194.18	57
Broussonin A	C_16_H_18_O_3_	258.31	57
Formononetin	C_16_H_12_O_4_	268.26	57
Senkyunolide A	C_12_H_16_O_2_	192.25	57
Paeoniflorin	C_23_H_28_O_11_	480.46	56
Atractylenolide III	C_15_H_20_O_3_	248.14	56
β-Sitosterol	C_29_H_50_O	414.71	55

**Table 2:** List of primer sequences.

Gene	Description	Accession No.		Sequence (5′-3′)
*TRAIL*	Tumor necrosis factor-related apoptosis-inducing ligand	NM_001024696	Forward	GCA GAC CTG TGT GTT GAT CC
			Reverse	GGG ATC CCA GAA ACT GTC AT
*BCL6*	B-cell CLL/lymphoma 6	XM_005657112	Forward	GTG TCC TAC GGT GCC TTT TT
			Reverse	TGA CGC AGA ATG TGA TGA GA
*FOXO3*	Fork headbox O_3_	NM_001135959	Forward	TCA GCC AGT CTA TGC AAA CC
			Reverse	CCA TGA GTT CGC TAC GGA TA
*CASP3*	Caspase 3	NM_214131	Forward	CTC AGG GAG ACC TTC ACA AC
			Reverse	GCA CGC AAA TAA AAC TGC TC
*GAPDH*	Glyceraldehyde-3-phosphate dehydrogenase	NM_001206359	Forward	ACA CCG AGC ATC TCC TGA CT
			Reverse	GAC GAG GCA GGT CTC CCT AA

## Data Availability

The original contributions presented in this study are included in this article. Further inquiries can be directed to the corresponding author.

## Abbreviations

*BCL6*	B-cell lymphoma 6
*CASP3*	Caspase 3
DON	Deoxynivalenol
*FOXO*	Forkhead box O
*TRAIL*	Tumor necrosis factor-related apoptosis-inducing ligand
DMEM	Dulbecco’s modified Eagle’s medium
FBS	Fetal bovine serum
PI	Propidium iodide
